# Use of multi-criteria decision analysis (MCDA) to support decision-making during health emergencies: a scoping review

**DOI:** 10.3389/fpubh.2025.1584026

**Published:** 2025-05-09

**Authors:** Stanislav Gaievskyi, Nicholas Delfrate, Luca Ragazzoni, Awsan Bahattab

**Affiliations:** ^1^Center for Research and Training in Disaster Medicine, Humanitarian Aid and Global Health, Università del Piemonte Orientale, Novara, Italy; ^2^Department for Sustainable Development and Ecological Transition, Università del Piemonte Orientale, Vercelli, Italy; ^3^Department of Translational Medicine, Università del Piemonte Orientale, Novara, Italy

**Keywords:** multi-criteria decision analysis, MCDA, health emergencies, decision support, scoping review

## Abstract

**Background:**

The mismatch between the health needs of populations affected by emergencies and resources devoted to response is projected to further increase. Making the response more effective is one of the solutions to meet the growing needs. Multi-criteria decision analysis (MCDA) has been successfully used to increase effectiveness in various fields by supporting decision-making. However, no review of its application to all-hazard health emergencies has been done to date.

**Methods:**

A review of peer-reviewed English-language articles published since 2004 was conducted in May 2024 using Scopus, PubMed and Web of Science databases. The review focused on the empirical application of MCDA to support decision-making during health emergencies. The review was guided by the Joanna Briggs Institute methodology for scoping reviews and adhered to the Preferred Reporting Items for Systematic Reviews and Meta-Analyses extension for Scoping Reviews. Quantitative data were analyzed using summary statistics and qualitative data were analyzed using content analysis.

**Results:**

Seventy-one articles were included after screening. The articles described the MCDA application to support a variety of decision problems related to health emergency management. However, the technique was mostly applied to infectious hazards management and only seldom to other hazards. The review also found a lack of standardized methodology for identifying alternatives and criteria, weighting, computation of model output, methods of dealing with uncertainty, and stakeholder engagement.

**Conclusion:**

The review provides an overview of the current use of the MCDA approach to support decision-making in health emergency management and informs areas of future development. The review emphasizes that while MCDA is already used for infectious hazards, it is underutilized for other types of health emergencies. Developing tailored MCDA approaches for health emergencies, including defining evaluation criteria and stakeholder engagement, may improve uptake of the technique and benefit the efforts to meet the growing health needs of the population affected by emergencies, https://osf.io/6kd5s/.

## 1 Introduction

In recent years, significant attention from health emergency researchers and practitioners has been devoted to pandemics, often overlooking other health emergencies. Unsurprisingly, COVID-19 alone caused over eight million deaths and the loss of one-tenth of the global economy (1, 2). Nevertheless, it is essential to keep an all-hazards perspective, acknowledging the considerable impact on human health caused by natural disasters and armed conflict and their frequent overlap with outbreaks of infectious diseases. In total, 399 disasters related to natural hazards were reported in 2023 (3). These events resulted in 86,473 fatalities, affecting almost a hundred million people and causing US$202,7 billion in losses. However, the total funding for the health sector in the Unaided Nations’ Global Humanitarian Overview was only 34% covered in 2024 and 42% in 2023, which indicates a considerable mismatch between the needs of the affected population and the resources available to meet those needs (4). Furthermore, the occurrence and scale of all of the above hazards are only expected to increase (5, 6). Thus, the mismatch between the health needs of the population affected by emergencies and the resources devoted to response will be further exacerbated.

Making emergency response more effective and efficient is one of the solutions to the above problem, and it has been the focus of the health emergency community for some time (7). While considerable improvements have been achieved in many areas of the national and international response, criticism of the current state of affairs continues to persist, with decision-making at its forefront (8). Not surprisingly, making rational, evidence-based decisions during a health emergency can be challenging due to the high uncertainty, scarce evidence and its often-conflicting nature, and lack of decision-makers’ ability to process the evidence, as well as pressure for them to act quickly (9, 10). Furthermore, the involvement of stakeholders in the decision-making process is also difficult in such a context. Maintaining transparency and accountability of the decision taken is another critical challenge.

Multi-criteria decision analysis (MCDA) is a set of techniques used to improve decision making process using structured, explicit approaches to decision involving multiple criteria (11). This set of techniques helps decision makers to define which criteria are relevant, importance attached to each criterion, systematically consider opinion of stakeholders and to use this information to assess available alternatives. Data that feed into MCDA can range from the informed opinions of stakeholders to objective quantitative data, depending on its availability. It has been successfully used to support decision-making in various fields, such as finance, engineering design and environmental management. It has proven to be a useful tool to increase consistency, transparency and legitimacy of decisions. In recent years, MCDA has been increasingly used in health care for a variety of decision objectives, ranging from health technology assessments to priority settings (12).

The MCDA has been used for health emergencies especially for prioritization of pathogens. For example, the MCDA approach is used at the global level by the World Health Organization for regular updates of the list of bacterial pathogens of public health importance and at regional and national levels by the United States Center for Disease Control and Prevention to conduct One Health Zoonotic Disease Prioritization Workshops (13, 14). It has seen further uptake to a broader range of decision objectives, beyond prioritization of pathogens, during the COVID-19 response. For example, it has been applied to prioritize patients (15), select public health interventions (16), prioritize locations to deliver interventions (17), among others. Such extensive use of MCDA during the COVID-19 response suggests that it may benefit a broader range of health emergency-related decisions. While several literature reviews looked into application of MCDA within health care in general (12), in One Health (18), and in COVID-19 (19); to the best of our knowledge, no reviews have investigated MCDA application for managing all hazard’s health emergencies. By taking a closer look at this experience, we hope to identify the strengths and weaknesses of the use of this approach in health emergencies and suggest future directions for the development of specific MCDA tools for health emergency management.

Thus, our review aimed to study how the MCDA methodology is used to support decision-making during health emergencies.

## 2 Materials and methods

### 2.1 Design

This study employed a scoping review methodology of peer-reviewed articles guided by the Joanna Briggs Institute Manual for Evidence Synthesis (20) and reported using the Preferred Reporting Items for Systematic Reviews and Meta-analyses Extension for Scoping Reviews (PRISMA-ScR) (21). The review protocol was registered with the Open Science Foundation before data extraction started (22).

### 2.2 Search strategy

A literature search was conducted using PubMed, Scopus, and Web of Science databases in May 2024. The search was focused on three main themes using the Boolean operator AND, namely:

- MCDA, specifying names of different techniques as defined in the International Society for Pharmacoeconomics and Outcomes Research’s MCDA Emerging Good Practices Task Force Reports 1 and 2 (11, 23);
- health emergencies, specifying different emergencies as defined by the World Health Organization’s classification of health hazards (24); and
- response.

Associated keywords, their synonyms, and differences in spelling were added to each theme using the Boolean operator OR. A separate search string was developed for each database, accounting for database-specific syntax. The detailed search strategy is available in Supplementary material A.

The search outputs were imported into the Zotero reference manager to remove duplicates. Then, the deduplicated citations were exported to Excel for screening. Two independent reviewers screened titles, abstracts and full texts based on defined inclusion/exclusion criteria. Any disagreements were resolved through discussion.

### 2.3 Eligibility criteria

Inclusion exclusion criteria described using the Problem, Interest, Context (PICo) framework presented in Table 1. To be included in the review, the article had to empirically address the *problem* of health emergency-related decision-making. For this review, we defined a health emergency as any event that negatively affects human health and requires immediate response beyond the health system’s routine capacities, such as infectious disease outbreaks, natural hazards, and conflicts (24). Studies focusing on decisions for broader emergency response without explicit mentioning of health, such as humanitarian logistics, flood or earthquake evacuations were excluded.

**Table 1:** PICo inclusion/exclusion criteria.

Criteria group	Inclusion	Exclusion
(P) Problem	Empirical studies aimed at supporting health emergency decision-making	Studies focusing on broader response, not specifically on health (humanitarian logistics, flood and earthquake response)
(I) Interest	Use of MCDA techniques, in particular, multi-attribute decision-making	Other non multi-attribute MCDA techniques, such as multi-objective decision-making Non-MCDA techniques or technique used is not described
(Co) Context	Decision related to health emergency prevention, preparedness, response and recovery	Studies focusing on decision-making not in the context of health emergency
Study characteristics	Empirical studies Written in English language Peered reviewed Last 20 years (2004–2024)	Systematic and non-systematic reviews Letters to the editors and commentary Dissertation and thesis Studies with no empirical application

The *interest* of our review was the use of multi-attribute decision-making MCDA techniques, as they are the most commonly applied MCDA techniques for decision support in health care (11, 12), mainly due to the simplicity of their application. While multi-objective decision-making MCDA techniques are widely used for optimal resource allocation and may play an important role in emergencies, especially humanitarian logistics (12), we considered them beyond the scope of this study due to their relatively more complex application, which may not be suitable for group deliberation that we thought is an important feature of any emergency specific MCDA tool. Furthermore, we excluded articles that did not provide sufficient information on the methods. To meet the inclusion criteria, the decision problem described in the article had to be addressed in the *context* of any stage of a health emergency, including prevention, preparedness, response and recovery.

Furthermore, the articles included are relevant to the following *study characteristics*. We limited our search only to peer-reviewed articles. An initial search of gray literature revealed that while recommendations on conducting MCDA are present in gray literature (25), the experience of their empirical application is rarely reported. We considered articles published in English in the last 20 years (Jan 2004 – May 2024). We excluded systematic and non-systematic reviews, letters to the editors and commentary, and dissertations and theses. Studies without empirical application were also excluded.

### 2.4 Data extraction

A data extraction tool was built in Excel and piloted on five articles. The data extraction fields were as follows: article characteristics, health emergency context (decision objectives, emergency type, emergency phase, level of application), details of alternatives and criteria used, weighting and aggregation techniques, methods to address uncertainty, stakeholder engagement, and reported strengths and limitations of the technique.

Two reviewers (SG, ND) performed data extraction. Upon the extraction, one reviewer (SG) conducted data cleaning, coding, and quality checks of the extracted data from all articles. Any discrepancies were resolved through discussion between the two reviewers.

### 2.5 Synthesis of studies

Quantitative data analysis was conducted in Excel using built-in formulas and pivot tables. The analysis focused on summarizing the MCDA application context and model characteristics. The analysis of the findings followed a deductive thematic approach, with the themes informed by the recommended steps for MCDA implementation (11). Where relevant, we compared the characteristics between studies focusing on different decision objectives, prioritization of locations, interventions, pathogens, patients, priority setting and others. Qualitative data on strengths and limitations was analyzed using content analysis. Furthermore, we grouped various criteria in the included studies into ten larger thematic categories to streamline analysis. We presented overall results using narrative descriptions accompanied by frequency measures where relevant. Granular summaries of criteria used for each thematic category can be found in the supplement.

## 3 Results

**Figure 1:**
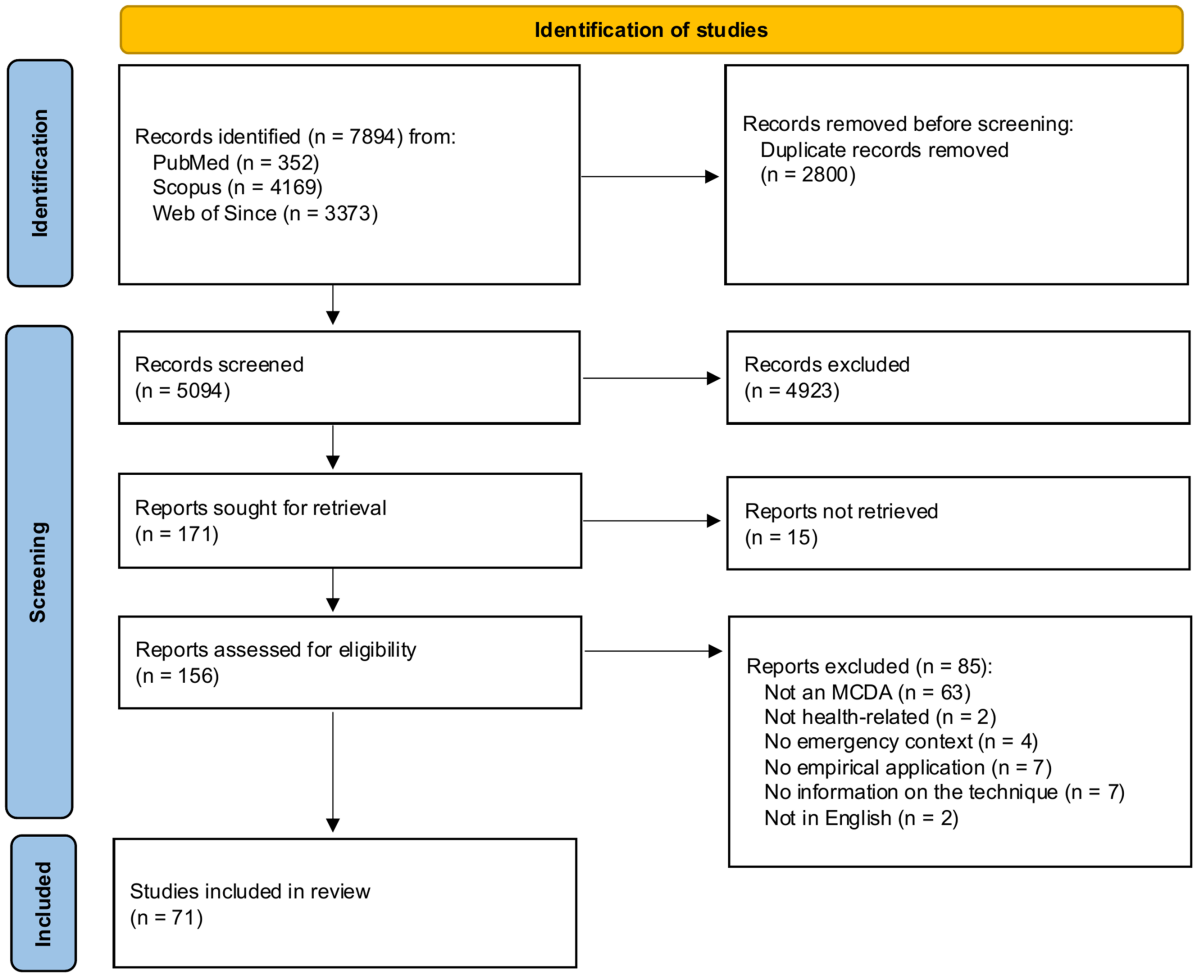
PRISMA flowchart for identification of studies.

A total of 7,894 citations were identified from the databases. After removing 2,800 duplicates, titles and abstracts of 5,094 citations were screened against the study inclusion/exclusion criteria, which resulted in the further exclusion of 4,923 citations. A total of 171 citations were sought for retrieval, of which 15 citations were not retrieved due to the lack of availability of the full text. Screening of full texts resulted in removing 85 citations, and 71 articles were included in the review. A PRISMA flowchart is presented in Figure 1. The list of all studies included in the review, along with summary statistics of key characteristics, is available in Supplementary material B.

### 3.1 Article characteristics

**Figure 2:**
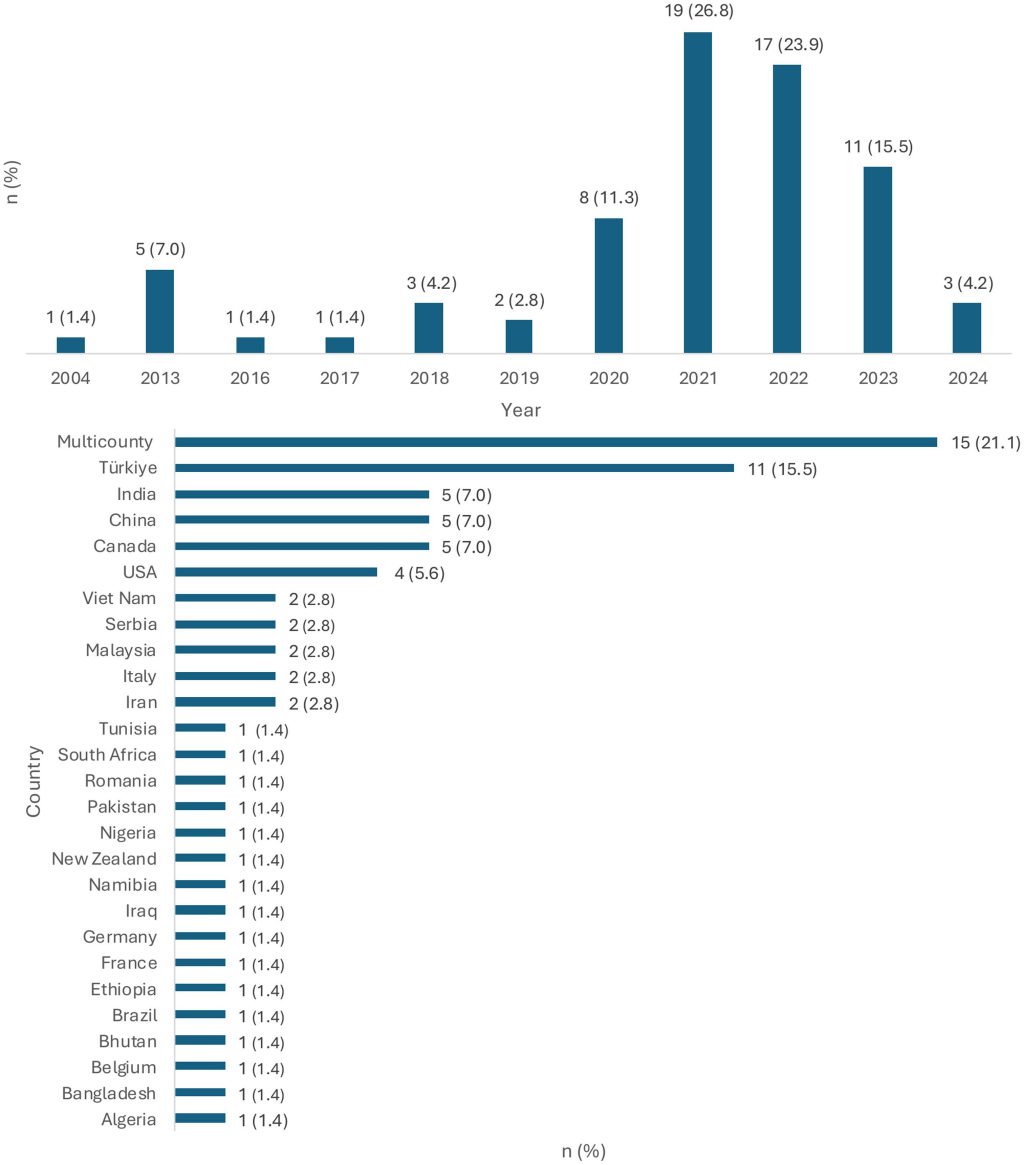
Characteristics of included articles **(A)** Publication year **(B)** Country of focus.

Among the included studies, the first study utilizing the MCDA approach for health emergencies was published in 2004. However, no studies meeting the review criteria were published between 2005 and 2013. The uptake of the methodology increased only at the beginning of 2020, with most studies included in the review (*n* = 55; 77.5%) being published between 2020 and 2023 (Figure 2).

In the review, over a fifth (*n* = 15; 21.1%) of the studies applied the methodology to multiple countries. Turkey (*n* = 11; 15.5%) was the most frequent country of focus of MCDA studies conducted, followed by China, Canada, and India (*n* = 5; 7.0% each).

### 3.2 Decision context

#### 3.2.1 Decision objective

A third of the included studies used MCDA methodology to prioritize locations (*n* = 21; 29.6%), with or without the use of Geo-Information System (GIS) tools (*n* = 9; 12.7% and *n* = 12; 16.9, respectively), followed by studies aimed at prioritizing health interventions (*n* = 17; 23.9%) and priority setting (*n* = 13; 18.3%) (Table 2). Studies prioritizing pathogens accounted for 12.7% (*n* = 9), and prioritization of patients for 9.9% (*n* = 4). Several studies focused on other decision objectives (*n* = 4; 5.6%), such as funding decisions (*n* = 2; 2.8%), selection of innovations and suppliers (*n* = 1; 1.4%, each).

**Table 2:** MCDA decision context.

Decision objective	*n*	%
Prioritization of locations	21	29.6
without GIS	12	16.9
with GIS	9	12.7
Prioritization of interventions	17	23.9
Priority setting	13	18.3
Prioritization of pathogens	9	12.7
Prioritization of patients	7	9.9
Other	4	5.6
funding	2	2.8
selection of innovations	1	1.4
supplier selection	1	1.4
Emergency type		
Infectious diseases	66	93.0
COVID-19	47	66.2
Multiple pathogens	9	13.6
Vector-borne diseases	5	7.0
Influenza-like viruses	3	4.2
HIV	1	1.4
Foodborne diseases	1	1.4
Multi-hazard (preparedness)	3	4.2
Natural hazard (earthquake)	1	1.4
Conflict (terroristic attack)	1	1.4

#### 3.2.2 Emergency type

Most studies focused on infectious diseases (*n* = 66; 93.0%), with COVID-19 being the leading disease. Only a fraction of the studies applied the methodology to multi-hazard emergencies (*n* = 3; 4.2%). Studies focusing on natural hazards and conflicts accounted for 1.4% (*n* = 1) each.

#### 3.2.3 Emergency phase

Most studies (*n* = 44, 62.0%) applied MCDA methodology during or post-emergency phase (response and recovery). However, some variation was observed between studies with different decision objectives, with priority setting mostly (*n* = 8; 61.5%) and prioritization of pathogens exclusively (*n* = 9; 100.0%) implemented in the pre-emergency context. At the same time, the prioritization of interventions (*n* = 13; 76.5%) and locations (*n* = 17; 81.0%) was mostly done in the post-emergency context and prioritization of patients only post-emergency (*n* = 7; 100.0%).

#### 3.2.4 Level of application

Studies that addressed decision objectives with a focus on the national level accounted for the majority of included studies (*n* = 26; 36.6%), followed by studies focused on sub-national (*n* = 19; 26.8%) and international levels (*n* = 15; 21.1%). Priority settings mainly were done at the international level (*n* = 7; 53.8%). On the other hand, prioritization of interventions was mainly done at the national level (*n* = 12; 70.6%) and prioritization of patients at the institutional level (*n* = 6; 85.7%).

### 3.3 Prioritization criteria and alternatives

The median number of criteria used in the selected studies for prioritization of interventions was 8 (range: 3–98; IQR: 5–12). With prioritization of pathogens using the highest number of criteria compared to other groups of studies (median: 13; range: 5–98; IQR: 9–24).

A literature review was the most common source for criteria identification, accounting for 39.4% (*n* = 28) of all included studies. It was mainly used for criteria identification in studies focusing on priority setting (*n* = 7; 53.8%) and prioritization of interventions (*n* = 9; 52.9%). A combination of literature review and expert consultations was used in 26.8% (*n* = 19) of all studies, with studies prioritizing locations (*n* = 7; 33.3%) and pathogens (*n* = 4; 44.4%) mainly reliant on this method. A notable proportion of studies did not provide information on how criteria were identified (*n* = 14; 19.7%).

Table 3 presents the criteria groups used in the study. Further breakdown of each group is available in Supplementary material C. Criteria that were considered characteristic of the population were the most frequently used criteria group among all included suites (*n* = 42; 18.6%); the second most used group of criteria was shared between the health system and societal characteristics, accounting for 13.7% each (*n* = 31). One-tenth of studies also used criteria related to cost/economic impact and environment and geography (*n* = 26; 11.5% and *n* = 25; 11.1%, respectively). Studies aimed at prioritization of interventions mostly relied on criteria related to society and cost/economic impact criteria groups (*n* = 13; 18.6% each), while studies prioritizing locations used environment and geography criteria groups most frequently (*n* = 16; 30.2%). Criteria related to ethics and equity were most prevalent in studies focusing on the prioritization of patients (*n* = 2; 20%).

**Table 3:** Criteria group by prioritization objective.

Criteria category	Priority setting		Interventions		Location		Pathogens		Patients		Other		Total	
	*n*	%	*n*	%	*n*	%	*n*	%	*n*	%	*n*	%	*n*	%
Population/BoD	8	22.9	5	7.1	12	22.6	9	20.5	7	70.0	1	7.1	42	18.6
Health system	8	22.9	7	10.0	7	13.2	6	13.6	1	10.0	2	14.3	31	13.7
Society	3	8.6	13	18.6	5	9.4	7	15.9	0	0.0	3	21.4	31	13.7
Cost/economic	2	5.7	13	18.6	4	7.5	5	11.4	0	0.0	2	14.3	26	11.5
Environment and geography	2	5.7	2	2.9	16	30.2	4	9.1	0	0.0	1	7.1	25	11.1
Threat characteristics	3	8.6	6	8.6	3	5.7	9	20.5	0	0.0	1	7.1	22	9.7
Feasibility	4	11.4	9	12.9	4	7.5	1	2.3	0	0.0	2	14.3	20	8.8
Ethics and equity	2	5.7	5	7.1	1	1.9	3	6.8	2	20.0	1	7.1	14	6.2
Effectiveness and safety	2	5.7	10	14.3	1	1.9	0	0.0	0	0.0	1	7.1	14	6.2
Global indexes	1	2.9	0	0.0	0	0.0	0	0.0	0	0.0	0	0.0	1	0.4

BoD, burden of disease.

The median number of alternatives used in prioritization was 13 (range: 1–775; IQR: 6–27). However, in studies using GIS, we did not count the number of alternatives that equated to the number of pixels on the map. Studies aimed at priority setting and prioritization of locations tended to include the greatest number of alternatives, with a median of 22 (range: 3–215; IQR: 11–30) and 20 (range: 3–775; IQR: 8.5–35.5), respectively. The least number of alternatives was considered in studies focusing on prioritization of interventions (median: 8; range: 3–27; IQR 4–15).

Among studies that implied the need for identification of alternatives, 40.5% (*n* = 17) explained how they were identified alternatives. Studies that did mention how alternatives were identified were almost equally split between identification from literature and consultations with stakeholders (*n* = 12; 28.6% and *n* = 10; 23.8% respectively); 7.1% (*n* = 3) identified alternatives combining literature review and expert consultations.

Over half of the included studies (*n* = 42; 59.2%) primarily used objective data to measure alternatives’ values. This was a prevalent source for value measurement in studies focusing on priority setting (*n* = 11; 84.6%), prioritization of location (*n* = 16; 76.2%) and patients (*n* = 6; 85.7%). At the same time, over one-third of all included studies (*n* = 25; 35.2%) used the opinion of stakeholders as a means for value measurement, which was the predominant source of data for the studies focused on prioritizing interventions and pathogens. Saaty’s (26) nine-point linguistic scale was the most commonly used scale for eliciting stakeholder opinion. Among all studies, only two studies (2.8%) combined objective data and opinions of stakeholders, both for prioritizing interventions.

### 3.4 Criteria weighting

Criteria weighting was used in almost all included studies (*n* = 69; 97.2%). No significant variation in use weighting was observed between studies focusing on different decision objectives. However, the included studies used a great variety of well-established and novel methods for criteria weighting, as well as combinations of different methods. The most used method was the Analytical Hierarchy Process (AHP), utilized in 38.6% (*n* = 27) of all studies. Objective rating and point allocation were the second most used methods for calculating weights, each accounting for 10% (*n* = 7) of all studies.

### 3.5 MCDA methods used

Similar to criteria weighting methods, the included studies employed various MCDA methods to compute the final model output. The weighted sum was the most used method overall, accounting for 28.2% (*n* = 20) of all studies; it was mainly used for the prioritization of locations and pathogens. The second most popular method was Technique for Order of Preference by Similarity to Ideal Solution (TOPSIS) (*n* = 9; 12.7%), predominantly used in studies that set priorities. A tenth of all studies did not report the MCDA method that they used.

### 3.6 Dealing with uncertainty

Uncertainty was addressed in two-thirds (*n* = 46) of all included studies. It was most frequently addressed in studies focused on the prioritization of pathogens (*n* = 8; 88.9%) and least frequently in studies prioritizing patients (*n* = 3; 42.9%).

The most frequently used method for addressing uncertainty was sensitivity analysis (*n* = 24; 52.2%), followed by fuzzy numbers (*n* = 10; 21.7%) and a combination of fuzzy numbers and sensitivity analysis (*n* = 7; 15.2%). Sensitivity analysis was primarily directed at testing output stability with changing weights. Several studies (*n* = 5; 10.9%) also used objective validation of the model result to address uncertainty.

### 3.7 Involvement of stakeholders

The majority (*n* = 61; 85.9%) of the studies involved stakeholders at one or several stages of the MCDA process. The median number of stakeholders involved was 12.5 (range 1–108), with the prioritization of pathogens involving the highest number of stakeholders (median: 37; range: 17–80) and the prioritization of patients the lowest (median: 3; range: 1–108).

Subject matter experts were the most frequently engaged group of stakeholders in all included studies (*n* = 52; 73.2%), followed by academics (*n* = 24; 33.8%) and government representatives (*n* = 21; 29.6%). Representatives of non-governmental organizations and affected community members were engaged only in a fraction of the studies (*n* = 4; 5.6% and *n* = 3; 4.2%, respectively). None of the included studies included donors/commissioners of the response to the MCDA process.

### 3.8 Strength and limitations

The reviewed studies identified several strengths and limitations of using MCDA in health emergency settings, namely, studies conducted by Bilal and İç (27) and Bouwknegt et al. (28) mentioned transparency, objectivity and reproducibility of results derived from the MCDA as its strengths. Another study, by Klamer et al. (29) reported MCDA’s ability to combine qualitative and quantitative data as another strength. Furthermore, Ekenberg et al. (16). emphasized the possibility of implementing the technique with imprecise evidence, and Guillot et al. (30) emphasized its flexibility and adaptability as an additional strength.

Another common strength mentioned in several articles, namely Hongoh et al. (31), Aenishaenslin et al. (32), and Thukral et al. (33), was the MCDA’s ability to meaningfully involve diverse stakeholders in decision processes. This reportedly led to an increase in the quality of the decisions taken and collaboration between stakeholders more broadly.

On the other hand, the included studies highlighted various limitations of using the MCDA for health emergencies. Bouwknegt et al. (28) reported that when objective data is limited and prioritization relies only on professional opinion, MCDA output can be subjective. Studies by Klamer et al. (29), Thukral et al. (33), and Nguyen et al. (34) emphasized that including only a small number of stakeholders or giving an unequal representation of different stakeholder groups involved in the prioritization exercises may also introduce potential biases. A further limitation of the method, highlighted in Hongoh et al. (31), is the time-consuming nature of the exercise, especially with elicitation methodologies requiring stakeholders to answer numerous pairwise comparison questions, which may make the engagement of necessary stakeholders challenging.

## 4 Discussion

The results provide insights into the types of emergencies where MCDA was applied, decision objectives it was aimed to support, approaches taken for identification of alternatives and criteria, methodologies for measurement of weights and performance, aggregation techniques, stakeholder engagement in the process, as well as strengths and limitations highlighted by the authors of the included studies.

The review showed that MCDA methodology can be applied to various decision objectives, ranging from prioritization of locations, health threats, and priority setting to selecting the best intervention to implement in response to an emergency. It can also be applied at different levels, starting from the facility, sub-national, national, and international levels, and used for decision objectives in anticipation of an emergency and after its occurrence.

The studies in the review highlighted that the MCDA also offers flexibility regarding methodology and data required for its deployment (16, 29, 30). It can be scaled depending on availability, time, and resource constraints. Furthermore, using the MCDA methodology increases transparency and accountability for decisions made during the response, potentially improving the response’s efficiency (31–33). This is a common quality of MCDA applications across contexts (18).

Our review demonstrated the initial application of MCDA methodology to support the management of health emergencies that occurred as early as two decades ago (35). After that, it was seldom used until a substantial increase happened during the COVID-19 response. While it proved to be a valuable tool for infectious disease management, especially for COVID-19, at national and international levels, it is underutilized for other types of health emergencies, such as natural disasters and conflicts, despite the listed advantages.

Our review demonstrated that standardized methodology across all stages of the MCDA process is lacking, except for MCDAs applied for prioritizing pathogens that follow somewhat standardized steps and methods. These findings are consistent with similar reviews that looked at the application of MCDA for other aligned health issues (12, 18, 19). Relatively low uptake among non-infectious disease emergencies can also be attributed to the lack of standard guidelines, as *ad hoc* methods must be developed for each exercise, increasing the time needed and requiring expertise. Furthermore, the absence of a standard approach limits the comparability and reproducibility of results and can increase biases. Thus, defining common MCDA steps and methods and a set of high-level criteria per emergency type or decision objective, drawing from successful MCDAs for disease prioritization, can reduce subjectivity and assist emergency managers in swiftly deploying the process in anticipation or shortly after an emergency.

The review also highlighted that stakeholders’ engagement in MCDA can positively influence the quality of the model output, as well as the broader coordination of stakeholders. Because the MCDA can involve a wide group of stakeholders with diverse views and systematically consider their preferences in the decision-making process. This finding coincides with another review by Zhao et al. (18) that highlighted a similar benefit of the MCDA process in the OneHealth field.

However, despite the clear benefits of stakeholder involvement in the MCDA, a standardized approach for selecting and engaging stakeholders is lacking, which may increase subjectivity and bias in the model outputs. Moreover, despite the opportunity to involve the population affected by emergencies in MCDA, which is in line with emergency response and humanitarian best practices, it is rarely done. Similarly, no explicit involvement of representatives of donors/commissioners of the response was observed in the MCDA studies included in our review. Including such a critical group of stakeholders can potentially strengthen trust and cooperation. Instead, the included studies seem to primarily rely on subject matter experts, who may provide a one-sided view of the situation. Therefore, if a specific MCDA tool for health emergency management were to be developed, it is essential to integrate standardized stakeholder engagement strategies.

Our study had several limitations. Firstly, we only included articles published in peer-reviewed journals in English, overlooking other languages and gray literature. Although we performed an initial scoping of gray literature and did not find any studies meeting predefined inclusion/exclusion criteria, conducting further searches with less stringent criteria and in different languages may reveal more information on the current state of MCDA use for health emergency management.

Secondly, we excluded studies that did not explicitly focus on health-related decision objectives, such as flood/tsunami/earthquake risk zoning, evacuations, and logistics of humanitarian supplies, such decision objectives also use MCDA techniques and studying them can provide further information for developing MCDA for health emergencies; however, decision objectives other than directly health-related were beyond the scope of this review.

Thirdly, the included studies used various MCDA techniques for weighting and aggregation, their combination, and some even proposed novel methods. Our review provided a high-level overview of those techniques and their practical application. However, additional analysis by researchers with mathematical expertise is warranted to identify the strengths and limitations of the used methods from a computational perspective.

In conclusion, the review provides an overview of the current use of the MCDA to support decision-making in health emergency management, which, to our knowledge, is the first review to investigate MCDA use in this context. The review emphasizes that while MCDA is already successfully used for specific decision objectives within some emergencies, such as infectious diseases prioritization, it is underutilized for other types of emergencies and decision objectives. Based on our review, we can suggest that to develop tailored MCDA for health emergencies, future research efforts should be directed toward:

- Defining best-suited MCDA implementation steps and methodology. Perhaps the work done by the MCDA Emerging Good Practices Task Force (11), along with insight from our article, may serve as the initial basis for this work.
- Defining a ‘catalogue’ of high-level prioritization criteria relevant to health emergencies that practitioners can draw upon, eliminating the need for repetitive development of *ad hoc* criteria. Thematic groups of criteria produced within this review can serve as a starting point.
- Establishing consensus on a standardized list of stakeholders that should be engaged in MCDA during emergencies, emphasizing the need for better involvement of representatives of affected communities as well as commissioners of the response, who are currently rarely included, as highlighted by our review.

Developing a tailored MCDA approach for all-hazard emergencies may improve uptake of the technique and benefit the efforts of emergency responders to meet the growing health needs of the population affected by emergencies.

## Supplementary material

SUPPLEMENTARY MATERIAL A Search string and number of citations per database.

SUPPLEMENTARY MATERIAL B List of all articles included in the review with summary table of key quantitative characteristics.

SUPPLEMENTARY MATERIAL C Breakdown of criteria thematic groups.
