# Supplementary material for: Use of multi-criteria decision analysis (MCDA) to support decision-making during health emergencies: a scoping review
Source: Front Public Health. 2025 May 9;13:1584026. doi: 10.3389/fpubh.2025.1584026 (PMC12098513; doi:10.3389/fpubh.2025.1584026)
Supplement: SUPPLEMENTARY MATERIAL A — Search string and number of citations per database. [file Table_1.DOCX]

*Supplement A: Search string and number of citations per database*

| **Database** | **Search string** | **Date** | **Number of citations** |
| --- | --- | --- | --- |
| PubMed | (("multi-criteri* decision*"[Title/Abstract] OR "multicriteri* decision*"[Title/Abstract] OR "multiple criteri* decision*"[Title/Abstract] OR "multicriteri* analysis"[Title/Abstract] OR "MCDM"[Title/Abstract] OR "MCDA"[Title/Abstract] OR "multi objective optim*"[Title/Abstract] OR "analytical hierarchy process"[Title/Abstract] OR "multi-attribute utility"[Title/Abstract] OR "weighted product method"[Title/Abstract] OR "technique for order preference by similarity to ideal solution"[Title/Abstract] OR "preference ranking organization method for enrichment evaluation"[Title/Abstract] OR "PROMETHEE"[Title/Abstract] OR "MACBETH"[Title/Abstract])  AND  ("health emergenc*"[Title/Abstract] OR "health hazard*"[Title/Abstract] OR "health crises*"[Title/Abstract] OR "health countermeasure*"[Title/Abstract] OR "pandemic*"[Title/Abstract] OR "epidemic*"[Title/Abstract] OR "outbreak*"[Title/Abstract] OR "infectious disease*"[Title/Abstract] OR "disaster*"[Title/Abstract] OR "earthquake*"[Title/Abstract] OR "flood*"[Title/Abstract] OR "tsunami*"[Title/Abstract] OR "storm*"[Title/Abstract] OR "drought*"[Title/Abstract] OR "wild fire*"[Title/Abstract] OR "war"[Title/Abstract] OR "conflict*"[Title/Abstract] OR "civil unrest*"[Title/Abstract] OR "terrorism"[Title/Abstract] OR "mass casualty incident*"[Title/Abstract] OR "MCI"[Title/Abstract])  AND  ("response"[Title/Abstract] OR "management"[Title/Abstract] OR "prioritisation"[Title/Abstract] OR "prioritization"[Title/Abstract] OR "priority setting"[Title/Abstract] OR "decision support"[Title/Abstract]))  AND ("2004"[Date - Publication] : "2024"[Date - Publication]) | 23 May 2024 | 352 |
| Scopus | ( TITLE-ABS-KEY ( "multi criteri* decision*" OR "multicriteri* decision*" OR "multiple criteri* decision*" OR "multicriteri* analysis" OR "MCDM" OR "MCDA" OR "multi objective optim*" OR "analytical hierarchy process" OR "multi-attribute utility" OR "weighted product method" OR "technique for order preference by similarity to ideal solution" OR "preference ranking organization method for enrichment evaluation" OR "PROMETHEE" OR "MACBETH" ) AND TITLE-ABS-KEY ( "health emergenc*" OR "health hazard*" OR "health crises*" OR "health countermeasure*" OR "pandemic*" OR "epidemic*" OR "outbreak*" OR "infectious disease*" OR "disaster*" OR "earthquake*" OR "flood*" OR "tsunami*" OR "storm*" OR "drought*" OR "wild fire*" OR "war" OR "conflict*" OR "civil unrest*" OR "terrorism" OR "mass casualty incident*" OR "MCI" ) AND TITLE-ABS-KEY ( "response" OR "management" OR "prioritisation" OR "prioritization" OR "priority setting" OR "decision support" ) ) AND PUBYEAR > 2003 AND PUBYEAR < 2025 AND ( LIMIT-TO ( DOCTYPE , "ar" ) ) | 20 May 2024 | 4169 |
| Web of since | (TS=("multi criteri* decision*" OR "multicriteri* decision*" OR "multiple criteri* decision*" OR "multicriteri* analysis" OR "MCDM" OR "MCDA" OR "multi objective optim*" OR "analytical hierarchy process" OR "multi-attribute utility" OR "weighted product method" OR "technique for order preference by similarity to ideal solution" OR "preference ranking organization method for enrichment evaluation" OR "PROMETHEE" OR "MACBETH")  AND  TS=("health emergenc*" OR "health hazard*" OR "health crises*" OR "health countermeasure*" OR "pandemic*" OR "epidemic*" OR "outbreak*" OR "infectious disease*" OR "disaster*" OR "earthquake*" OR "flood*" OR "tsunami*" OR "storm*" OR "drought*" OR "wild fire*" OR "war" OR "conflict*" OR "civil unrest*" OR "terrorism" OR "mass casualty incident*" OR "MCI")  AND  TS=("response" OR "management" OR "prioritisation" OR "prioritization" OR "priority setting" OR "decision support"))  AND  PY=(2004-2024)  AND TS=("health emergenc*" OR "health hazard*" OR "health crises*" OR "health countermeasure*" OR "pandemic*" OR "epidemic*" OR "outbreak*" OR "infectious disease*" OR "disaster*" OR "earthquake*" OR "flood*" OR "tsunami*" OR "storm*" OR "drought*" OR "wild fire*" OR "war" OR "conflict*" OR "civil unrest*" OR "terrorism" OR "mass casualty incident*" OR "MCI")  AND PY=(2004-2024) | 24 May 2024 | 3373 |
