# Supplementary material for: Use of multi-criteria decision analysis (MCDA) to support decision-making during health emergencies: a scoping review
Source: Front Public Health. 2025 May 9;13:1584026. doi: 10.3389/fpubh.2025.1584026 (PMC12098513; doi:10.3389/fpubh.2025.1584026)
Supplement: SUPPLEMENTARY MATERIAL C — Breakdown of criteria thematic groups. [file Table_3.DOCX]

Supplementary Material C: Breakdown of Criteria Thematic Groups

1. **Population / Burden of disease**
   1. Burden of disease
      1. Number/rate of
         1. cases
         2. fatalities
         3. recovered
         4. susceptible population
         5. global trends over 5 years
      2. Food-borne diseases
      3. Mental health
      4. Malaria cases among children
      5. Number of cases in animals
      6. Maternal mortality ratio
      7. Presence of disease in the region
   2. Population characteristics
      1. High health-risk populations (including occupation-related risks)
      2. Belonging to underserved minorities
      3. Immunization status
      4. No health insurance
      5. Smokers
   3. Clinical characteristics
      1. Age
      2. Sex
      3. BMI
      4. Blood pressure
      5. SpO2
      6. X ray finings
      7. Modified Early Warning Score-MEWS (Subbe et al., 2001)
      8. Laboratory examinations
         1. C-reactive protein
         2. white blood cell count
         3. neutrophil count/ratio
         4. lymphocyte count/ratio
         5. eosinophils count/ratio
         6. hemoglobin
         7. albumin
         8. interleukin
      9. Patient’s comorbidities
         1. diabetes
         2. obesity
         3. immune deficiency or immunosuppression
         4. renal failure
         5. chronic pulmonary diseases
         6. chronic cardiovascular diseases
         7. evolutive cancer
         8. bone marrow or organ transplantation
2. **Health system**
   1. Health system ability to detect and treat
      1. Capacity to detect
      2. Availability of medical countermeasures
      3. Existence and effectiveness of treatment
      4. Level of scientific knowledge of the disease
   2. Health system capacity
      1. Number/rate of
         1. tests
         2. nurses/doctors
         3. pharmacists
         4. infected/dead health workers
         5. beds/ICU beds
         6. ventilators
      2. Health care access
      3. Health workers’ experience with infectious diseases
      4. Health workers’ interest, attitude and communication style
      5. Risk perception of health workers
3. **Societal impact**
   1. Impact on
      1. Specific industries
      2. Access to education
      3. Trade
   2. Public attitudes
      1. Risk perception
      2. General level of knowledge, attitude and behavior of the public
      3. Risk perception of decision-makers
   3. Venerability
      1. Percentage of population comprising lowest wealth quintile
      2. Internally displaced populations
      3. Child poverty
      4. Unemployed
      5. Senior citizen (above 60 age group)
      6. Low food access
      7. No private vehicle availability
      8. Population living in slums
   4. Education level
      1. Literacy rate
      2. High school graduation rate
   5. Preventative measures
      1. Number of years since last mass insecticide-treated bed nets distribution
      2. Proportion of households with at least 1 insecticide-treated bed nets per 2 people
   6. Population density and mobility
      1. Household size
      2. Urban male population
      3. Urban population
      4. Working-age population
      5. Spatial interaction index
   7. Water, sanitation and hygiene availability
      1. Sanitation
      2. Drinking water
      3. Hygiene
   8. Border health
      1. Air transport passengers carried
      2. International tourism number of arrivals
      3. Points of entry
   9. Other
      1. Resilience
      2. Access to cities
      3. Mobile cellular subscriptions
      4. Internet users
      5. Road density
4. **Cost / economic**
   1. Short-term costs to
      1. Provincial government
      2. Private sector
      3. Individuals
   2. Health expenditure
      1. Public and private expenditure on health care
      2. Current health expenditure
      3. Socioeconomic impact (including on vulnerable communities)
5. **Environment and geography**
   1. Population density
      1. Population density
      2. Built-up area presence index (proxy for urban/rural designation)
      3. Total length of roads in the region
      4. Total length of railways in the region
   2. Distances infrastructure (to/from)
      1. Roads
      2. Hospitals
      3. Bus stations/terminals
      4. Banks
      5. Markets
      6. Health care unit
      7. Government offices
   3. Environmental
      1. Mean annual rainfall
      2. Temperature suitability index
      3. Land surface temperature
      4. Landfill sites
      5. Water logging
      6. Land use
   4. Other
      1. Zones where tick populations present/established
      2. Existence of favorable conditions for disease transmission
      3. Influence of climate change
6. **Pathogen characteristics**
   1. Introduction probability
   2. Human transmission
   3. Evolutionary potential
   4. Congenital risk
   5. Reportable disease (nationally or internationally)
   6. WHO objective for eradication
   7. Level of scientific knowledge of the disease
   8. Existing antibiotic multidrug resistance
   9. Reference laboratory essential for diagnosis
   10. Connections with biological weapons programmes
   11. High risk of occupational exposure
   12. Vaccine included in national vaccination programme
   13. Human/animal interface
   14. Severity or case fatality rate in humans and in animals
   15. Cumulative attack rate (that is the proportion of the statewide population infected during the pandemic, and a simulated outcome from the model)
   16. Ability to infect environment
7. **Feasibility**
   1. Implementation complexity
   2. Delay before results
   3. Level of public acceptance
   4. Government effectiveness
   5. Risk of governmental abuses
   6. Optimization opportunities
   7. Existing inter-sectoral collaboration for surveillance and reporting
8. **Ethics and equity**
   1. Potential to increase social inequality
   2. Population living with vulnerable people (i.e. people with comorbidities, pregnant women, or immunosuppressed patients)
   3. Proportion of population benefitting from intervention
   4. Human rights
   5. Vulnerable groups
9. **Effectiveness and safety**
   1. Reduction in incidence of human cases
   2. Reduction in entomological risk
   3. Impacts of adverse health effects
   4. Impact on wildlife
   5. Impact on organization’s credibility
10. **Global Indexes**
    1. Stringency index
    2. Human development index
    3. Operational readiness index
    4. Corruption perception index
    5. INFORM COVID-19 index
